# DIA-based quantitative proteomics explores the mechanism of amelioration of APAP-induced liver injury by anoectochilus roxburghii (Wall.) Lindl

**DOI:** 10.3389/fphar.2025.1508290

**Published:** 2025-03-26

**Authors:** Wenjie Dong, Yao Mou, Qiuyu Li, Min Li, Hao Su, Longyang Jiang, Jie Zhou, Kun Tu, Xuping Yang, Yuexi Huang, Changjing Xu, Liaoyun Zhang, Yilan Huang

**Affiliations:** ^1^ Department of Pharmacy, The Affiliated Hospital, Southwest Medical University, Luzhou, China; ^2^ School of Pharmacy, Southwest Medical University, Luzhou, China; ^3^ Department of Critical Care Medicine, The Affiliated Hospital, Southwest Medical University, Luzhou, China; ^4^ Department of Pharmacy, Sichuan Provincial Woman’s and Children’s Hospital, The Affiliated Women’s and Children’s Hospital of Chengdu Medical College, Chengdu, China

**Keywords:** drug-induced liver injury, acetaminophen, anoectochilus roxburghii (Wall.), polysaccharides, oxidative stress and inflammation

## Abstract

**Background:**

Drug-induced liver injury (DILI) is the most common cause of acute liver injury. Anoectochilus roxburghii (Wall.) Lindl. (AR) and its polysaccharide fractions (ARPs) have been shown to have effective therapeutic effects with minimal side effects on a wide range of diseases including hepatopathy. This study aims to determine the therapeutic effects of ARPs on acetaminophen (APAP)-induced liver injury and to explore the mechanistic pathways involved.

**Methods:**

C57BL/6J male mice at 8 weeks were used to construct a model of APAP-induced liver injury. The acute hepatic injury was induced by oral administration of APAP (300 mg/kg) before 16 h fasting. For therapeutic experiment, mice were gavaged with the water extract of AR (AR.WE) or the purified ARPs before and after APAP administration. Biochemical analyses, ELISA analyses, H&E staining, RT-PCR, and Quantitative proteomic analysis were used to investigate the effects and mechanisms of AR on DILI.

**Results:**

Both AR.WE. and the purified ARPs treatment reduced APAP-induced liver injury, decreased hepatic glutathione and TNF-α levels, alleviated oxidative stress and inflammation. Quantitative proteomic analysis revealed that ARPs downregulated the protein levels involved in apoptosis, inflammation, oxidative stress, necroptosis, while upregulated the protein levels involved in autophagy. These protective effects of ARPs are possibly related to the downregulation of vATPase activity and thus participating in the autophagic process and ferroptosis.

**Conclusion:**

ARPs can protect mice against APAP-induced liver injury, alleviate oxidative stress and inflammation. Our study reveals a potential therapeutic effect for ARPs in protecting APAP-induced liver injury.

## 1 Introduction

Drug-induced liver injury (DILI) is the most common cause of acute liver injury (Kumachev and Wu, 2021). In the USA, more than 50% of all cases of acute liver failure are attributed to DILI, among which idiosyncratic DILI accounts for over 10% of the reported cases (Li et al., 2022). Acetaminophen (APAP) is one of the most commonly used antipyretic and analgesic drugs (Yan et al., 2018) and the Food and Drug Administration has recommended a maximum daily dose of 4 g per day for adults (Orandi et al., 2023). Today, overdose of APAP is the leading cause of pharmacological acute liver injury worldwide (Lee, 2017; Reuben et al., 2016). In the body, most of the APAP is metabolized to nontoxic compounds that are ultimately excreted out of the body via the kidneys or bile, and no more than 10% of APAP is activated by cytochrome P450 enzymes (mainly CYP2E1) to an active product (n-acetyl-p-benzoquinone-imine (NAPQI)) (Chiew et al., 2023; McGill et al., 2012), which binds to glutathione (GSH) to deplete the amount of glutathione in the liver (Cai et al., 2022). In addition, binding of NAPQI to intracellular proteins in the hepatocyte leads to mitochondrial dysfunction and ultimately to hepatocyte necrosis (Yan et al., 2018). To date, n-acetylcysteine (NAC) is the only antidote used for APAP poisoning. However, its therapeutic timeframe is narrow (it is effective only within 12 h of APAP poisoning) (Chowdhury et al., 2020; Licata et al., 2022). Therefore, there is an urgent need to find new targets as well as to develop new drugs for clinical treatment to improve survival.

In recent years, with the in-depth study of traditional Chinese medicines, some of them have been found to have detoxifying and hepatoprotective effects, provide unique advantages in the treatment of liver injury. Zhou et al. found that baicalein could ameliorate APAP-induced liver injury by a mechanism involving the Jak2/Stat3 and MAPK signaling pathways (Zhou et al., 2018). Meanwhile, Jing et al. found that celastrol-loaded biomimetic nanodrug ameliorated APAP-induced liver injury by modulating macrophage polarization (Zheng et al., 2023). In addition, Zhong et al. found that ginsenoside Rc treatment prevented inflammatory responses induced by APAP and oxidative stress in primary mouse hepatocytes and corresponding changes in related genes (Zhong et al., 2022). Recently, in a comprehensive review of clinical trials of natural products as hepatoprotective agents, it was noted that Chinese herbal medicines such as berberine, turmeric, schizandra, and silymarin, as well as natural products such as artichoke, chlorella, and spirulina, improved some measures of liver outcomes after treatment of liver injury (Służały et al., 2024). Therefore, there is a need to explore more potential agents for hepatic injury treatment, and natural products derived from traditional Chinese herbal medicines may provide alternative treatment options for liver injury.

Anoectochilus roxburghii (Wall.) Lindl. (AR), a traditional herbal medicine belonging to the family of Orchidaceae, is widely distributed in Southeast Asia (Cui et al., 2023). The polysaccharides of AR polysaccharides (ARPs) are the main water-soluble constituents of the plant, which have various pharmacological activities such as improvement of immunity, glycemic control, blood circulation, and anti-oxidation (Qiu et al., 2023). Previously, our research group found that the ARPs treatment can inhibit hepatic lipid deposition and liver steatosis, increase fatty acid oxidation, thereby improving liver lipid metabolism in diet-induced fatty liver (Fu et al., 2022; Tian et al., 2022). Similarly, Huang et al. found that AR oral liquid exerted a protective effect against alcoholic liver injury in rats by attenuating oxidative stress and inflammatory infiltration (Huang et al., 2023). In addition to this, it has been found that ARPs can reduce serum ALT and AST by decreasing oxidative stress and inhibit the production of hepatic malondialdehyde in a mouse model of carbon tetrachloride-induced acute hepatic injury (Yang et al., 2017). However, whether AR is effective against acute liver injury caused by acetaminophen is less clear. In the present study, we found that aqueous extracts of AR and its polysaccharide fractions ARPs has preventive and therapeutic effects on acute liver injury caused by APAP, the hepatoprotective effect of this herb is at least partially connected with hepatocyte autophagy.

## 2 Methods

### 2.1 Preparation of AR.WE and ARPs

Medicinal herbs of AR was purchased from Sichuan Tianzhi Co. The extraction method was performed according to the previous study of our research group (Fu et al., 2022). Briefly, AR.WE was prepared with distilled water (1:50, w/v), and the ARPs fractions were collected by water extraction and alcohol precipitation. The polysaccharide content was determined by the phenol-sulfuric acid method (sulfuric acid: phenol: polysaccharide = 5:1:2). Concentrated sulfuric acid (95%) was added and left in a water bath at 100°C for 10 min and the absorbance was measured at 490 nm. Finally, for further study, the precipitate was washed with distilled water and freeze-dried. The ARPs fractions were identified by High-performance liquid chromatography (HPLC, Agilent, Palo Alto, CA, United States) and separated on a Shiseido C18 column (4.6 mm × 250 mm, 5 μm, pore size). The molecular weights of the fractions were determined by gel permeation chromatography (GPC) on a Viscotek GPCmax VE2001 (Malvern Instruments, UK) chromatograph.

### 2.2 Animal

C57BL/6J male mice, 6–8 weeks old were purchased from HFK Biological Technology Co. Ltd, and all mice were housed in standard procedures in the Animal Experiment Center of Southwest Medical University. They were kept in the Animal Experiment Center of Southwest Medical University with 12 h of light and dark cycles, 20°C–25°C, 50%–70% humidity, and free access to food and water. For ALIL model construction, APAP was dissolved in lukewarm water at 50°C and used as it was prepared. In this study, the drug was administered by gavage. In the prevention experiment, a total of 18 C57BL/6J mice were gavaged with AR.WE (250 mg/kg) (n = 6) or ARPs (250 mg/kg) (n = 6) for three consecutive days, and on the last day, the mice were fasted for 16 h and then given 300 mg/kg APAP to induce acute liver injury. In the treatment experiments, mice were gavaged with 300 mg/kg APAP to induce acute liver injury after 16 h of fasting. One hour later, mice were gavaged with AR.WE. (250 mg/kg) (n = 6). Ten hours after administration, mice were anaesthetised with isoflurane and blood and liver were taken for testing. All experimental designs were approved by the Ethics Committee of the Animal Experiment Center of Southwest Medical University.

### 2.3 Cell culture and treatments

HepG2 cells were cultured using medium containing 10% FBS and 1% penicillin-streptomycin and were incubated at 37°C in an incubator containing 5% CO_2_. After pretreatment with ARPs (0.5 μg/mL and 1 μg/mL) for 1 h, APAP (2.5 mM) was given for stimulation for 24 h before subsequent experiments.

### 2.4 Plasma AST and ALT levels

Blood was taken from mice after anesthesia and execution, and whole blood samples were centrifuged at 4°C at 6,000 rpm for 10 min, the supernatant was taken in a new tube. Next, the supernatant was centrifuged again at 4°C at 6,000 rpm for 10 min to remove impurities, and then plasma was collected. Plasma was stored in a refrigerator at 80°C and assayed with ALT and AST kits (Zhongsheng Technologies, China). 200 μL of assay solution was added to 5 µL of serum/standard/PBS (blank control) and immediately subjected to enzyme kinetic assay at 37°C.

### 2.5 Hepatic GSH detection

The surface of mouse liver was washed with PBS, then added with saline homogenate, centrifuged at 4°C, 4,000 rpm for 10 min, and the supernatant was taken for measurement. The GSH content in the supernatant was detected using a GSH kit (Nanjing Jiancheng, China).

### 2.6 Hepatic SOD detection

The surface of mouse liver was washed with PBS, then added with saline homogenization, centrifuged at 4°C, 4000rpm for 10 min, and the supernatant was taken for measurement. The SOD content in the supernatant was detected by using SOD kit (Nanjing Jiancheng, China).

### 2.7 H&E staining

The same leaf of mouse liver was taken and immediately fixed in 4% paraformaldehyde, then the dehydrated liver tissue was embedded in paraffin, and the tissue wax block was cut into sections of about 4 μm thickness. Each section was dewaxed, rehydrated and stained with hematoxylin and eosin. Microscope equipped with a DFC350FX digital camera (Leica, Milano, Leica) was used to observe the tissue morphology.

### 2.8 Enzyme-linked immunosorbent assay (ELISA)

Whole blood samples were centrifuged at 4°C, 6,000 rpm for 10 min, and the supernatant was taken in a new tube next, and the supernatant was centrifuged again at 4°C, 6,000 rpm for 10 min to remove impurities, and then plasma was collected. The plasma was stored in a refrigerator at 80°C, and the concentration levels of serum IL-6, TNF-α (ml002095, mlbio, China) were determined using ELISA kits according to the manufacturer’s instructions. Add 10 µL of biotin labelled antibody and 50 µL of enzyme reagent to 40 µL of serum/standard/PBS. Warm bath at 37°C for 30 min and then washed with washing solution. Finally the absorbance was measured after adding the colour developer.

### 2.9 Quantitative real-time polymerase chain reaction analyses (RT-PCR)

Total RNA from liver and HepG2 cells were extracted using TRIzol reagent (Vazyme, Nanjing, China), and 1,000 ng of RNA was reverse transcribed to complementary DNA using a PrimeScript RT Reagent Kit (Takara RR037A). 1,000 ng of RNA was extracted using SYBR^®^ Green Realtime PCR Master Mix (Novizan, Nanjing, China) was used to perform real-time quantitative PCR on a Bio-Rad CFX ConnectTM Real-Time system (Bio-Rad, United States). The following procedure was used: pre-denaturation at 95°C for 5 min, denaturation at 95°C for 20 s, annealing at 60°C for 20 s, and extension at 72°C for 20 s, for a total of 39 cycles. The relative gene expression levels were assessed using the 2^−ΔΔCT^ method with 18s or GAPDH as control. Primer sequences are shown in Supplementary Table S1.

### 2.10 Analysis of DIA-based quantitative proteomics

#### 2.10.1 Protein extraction

Liver samples were first grinded by liquid nitrogen and then the powder was transferred to a 1.5 mL centrifuge tube and sonicated three times on ice, using a high intensity ultrasonic processor in a lysis buffer (8M urea including 1mM PMSF, 2 mM EDTA). Then, the remaining debris was removed by centrifugation at 15,000 g at 4°C for 10 min. Finally, the protein concentration was determined with a BCA kit according to the instructions of the manufacturer.

#### 2.10.2 Digestion and cleanup

Equal amount of proteins from each sample were used for tryptic digestion. Add 8M urea to 200ul to the supernatants, then reduced with 10 mM DTT for 45 min at 37°C and alkylated with 50 mM iodoacetamide (IAM) for 15 min in a dark room at room temperature. 4×volume of chilled acetone was added and precipitated at −20°C for 2 h. After centrifugation, the protein precipitate was air-dried and resuspended in 200ul of 25 mM ammonium bicarbonate solution and 3 μL of trypsin (Promega) and digested overnight at 37°C. After digestion, peptides were desalted using C18 Cartridge followed by drying with Vacuum concentration meter, concentrated by vacuum centrifugation and redissolved in 0.1% (v/V) formic acid.

#### 2.10.3 LC-MS/MS analysis

LC-MS/MS analysis was performed on an Orbitrap Astral MS coupled to a Thermo ScientificTM VanquishTM Neo UHPLC system, and interfaced online using an EASYSprayTM Nano source. Sample was injected via autosampler and trapped on an PepMap Neo Trap Cartridge column (300 μm × 5 mm, 5 μm), and then separated on an Easy-SprayTM PepMapTM Neo UHPLC column (150 μm × 15 cm, 2 μm) with a gradient time of 6.9 min. For the DIA experiments, the Orbitrap Astral MS was operated at a full MS resolution of 240,000 at 200 m/z with a full scan range of 380–980 m/z when stated. The full MS AGC was set to 500%. Fragmention scans were recorded at a resolution of 80,000 and Maximum lnjection Time (ms) of 3 ms. 299 windows of 2-Th scanning from 380 to 980 m/z were used. The isolated ions were fragmented using HCD with 25% NCE.

#### 2.10.4 Database search and quantification

MS raw data were analyzed using DIA-NN (v1.8.1) with library-free method. The uniprot-proteome_UP000000589_Mus_musculus.fasta database (A total of 55,319 sequences), iRT2.fasta database (A total of 1 sequences) was uesed to creat a spectra library with deep learning algrithms of neural networks. The option of MBR was employed to create a spectral library from DIA data and then reanlyse using this library. FDR of search results was adjusted to <1% at both protein and precursor ion levels, the remaining identifications were used for further quantification analysis.

### 2.11 Data and statistical analysis

Besides the data from DIA-based quantitative proteomics, other data were statistically analyzed using Graph Pad Prism 9.5 software (San Diego, CA, USA). Student's t-test was used for comparisons between two groups. Comparisons between multiple groups were analyzed using one-way ANOVA, and *post hoc* test adjustments were made using Bonferroni correction. Post hoc tests were performed only when F reached *p* < 0.05 and there was no significant heteroscedasticity.

## 3 Results

### 3.1 AR.WE mitigated APAP-induced liver injury in the prevention experiments

**FIGURE 1:**
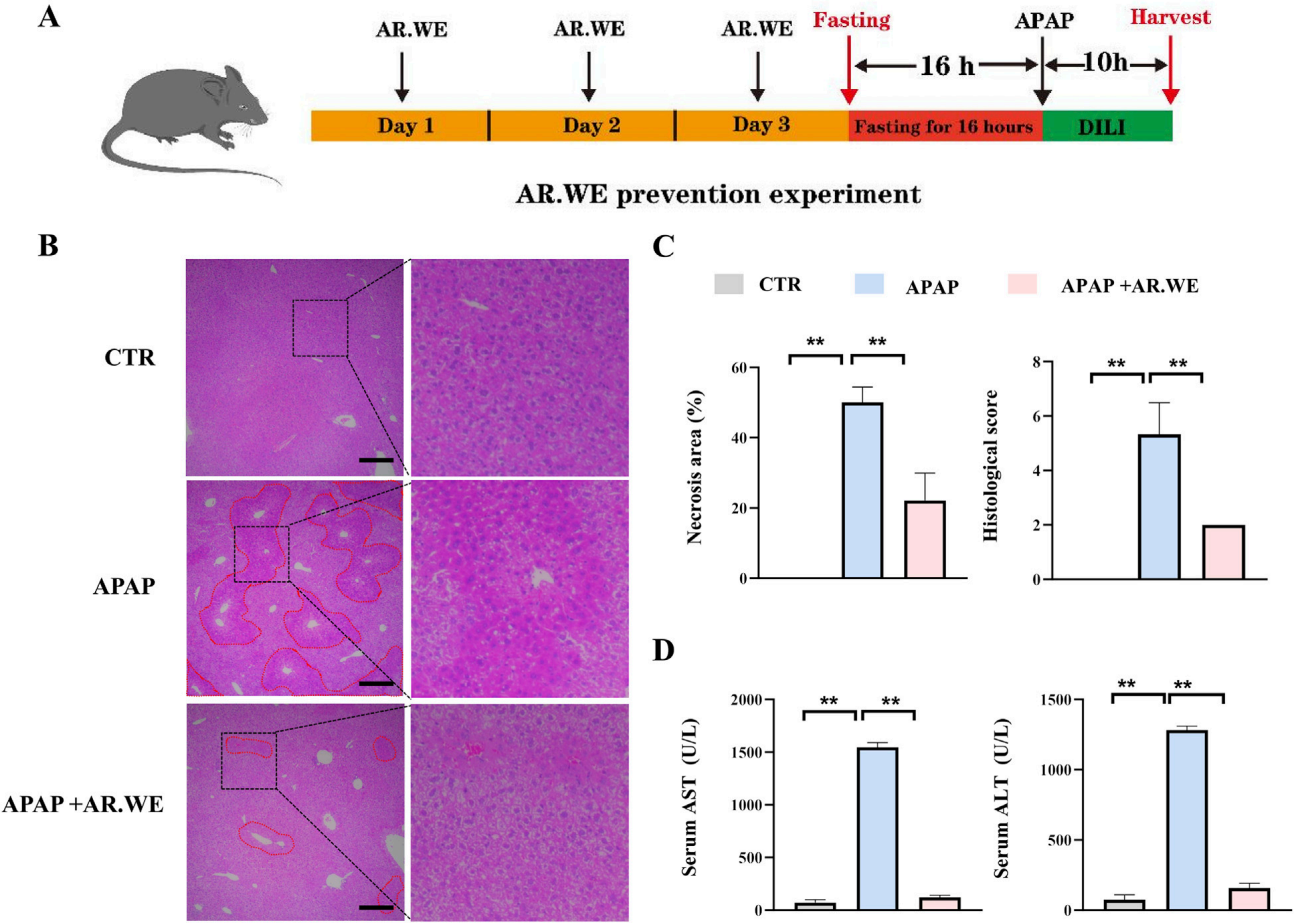
Anoectochilus roxburghii water extract (AR.WE) can prevent hepatotoxicity induced by APAP. **(A)** The time schedule for the AR.WE for the prevention of APAP-induced liver injury. Mice were gavaged with AR.WE (250 mg/kg) for 3 days continuously, and fasting was initiated for 16 h after the end of gavage on the third day. Acute liver injury was induced by administration of 300 mg/kg APAP at the end of fasting. Then, blood and liver samples were collected after 10 h **(B)** H&E staining of mouse liver sections after prophylaxis with AR.WE The dotted line indicates central lobular necrosis. Scale bars, 100 μm, n = 3. **(C)** Percentage of necrotic areas (left) and liver histological scores (right), n = 3 **(D)** Serum ALT (left) and AST (right) levels. All group data subjected to statistical analysis were repeated in at least three independent experiments, n = 5–6. The data are presented as mean ± SEM. *p < 0.05, **p < 0.01.

APAP-induced liver injury is mainly characterized by histopathological changes and hepatic necrosis (Chen et al., 2023). We assessed the preventive effects of AR.WE on acute liver injury induced by APAP using a dose of 250 mg/kg (Figure 1A). The hallmark of APAP-induced liver injury is centrilobular liver necrosis (Yan et al., 2018). H&E results showed that pretreatment of AR.WE significantly reduced pathological changes and necrotic area of mouse liver (Figures 1B,C). Furthermore, ALT and AST expression in serum AST and ALT are specific aminotransferases in the liver. When hepatocytes are damaged, ALT and AST are released in large amounts into the blood, and serum ALT and AST levels are increased (Lv et al., 2019). APAP-treated mice showed high ALT and AST levels in the blood test, the biochemical outcomes demonstrated that the AR.WE pretreatment considerably decreased the blood ALT and AST levels induced by APAP (Figure 1D).

**FIGURE 2:**
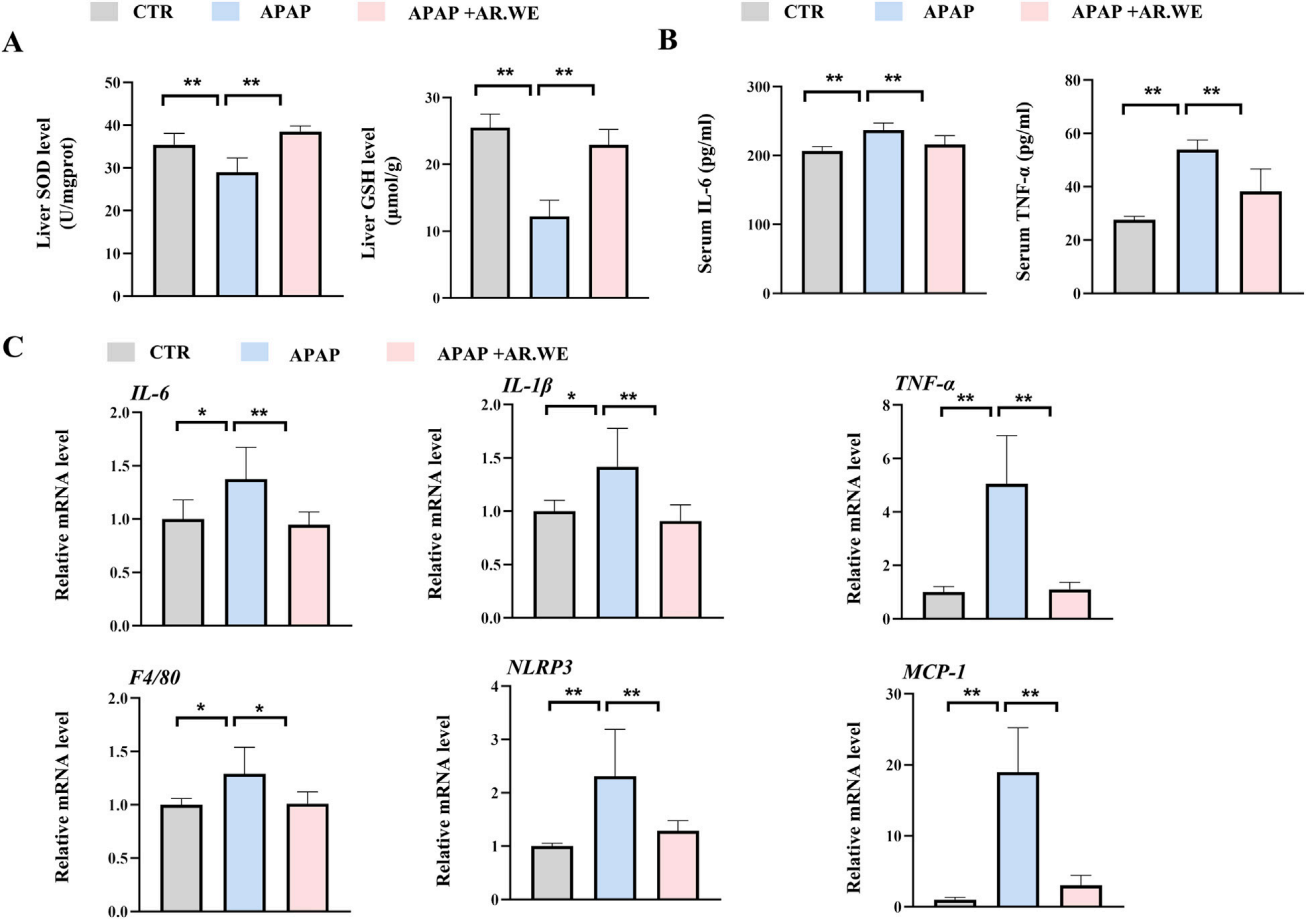
AR.WE can prevent APAP-induced inflammation and oxidative stress. **(A)** ELISA analysis of serum IL-6 (left) and TNF-α (right), n = 6. **(B)** The level of liver SOD (left) and GSH (right), n = 5–6 **(C)** RT-PCR analysis of *IL-6, IL-1β, TNF-α, F4/80, NLRP3, MCP-1*, n = 5–6. All group data subjected to statistical analysis were repeated in at least three independent experiments. The data are presented as mean ± SEM. *p < 0.05, **p < 0.01.

Superoxide Dismutase (SOD) is an important antioxidant enzyme that clears harmful free radicals. APAP-induced liver injury often leads to reduced SOD levels, which triggers oxidative stress in the body (Li et al., 2023a). However, we found that AR.WE pretreatment significantly increased hepatic SOD levels, thereby reducing the occurrence of oxidative stress (Figure 2A). Meanwhile, Glutathione (GSH) depletion is a critical step in APAP-induced liver injury. We detected variations in liver GSH activity in mice. The results demonstrated that APAP significantly reduced the activity of hepatic GSH compared to the control group, whereas AR.WE preconditioning significantly increased the activity of hepatic GSH (Figure 2A). APAP-induced liver injury is often accompanied by a powerful inflammatory response (Xu L. et al., 2022). To assess the inflammatory state in the liver, enzyme activity and protein expression levels of a certain factor involved in the inflammatory pathway were detected. We examined the serum pro-inflammatory cytokine protein and mRNA levels such as TNF-α by using ELISA analysis (Figure 2B) and RT-PCR analysis (Figure 2C), respectively. APAP significantly increased serum IL-6 and TNF-α levels and expression of genes involved in inflammation compared to controls. As expected, AR.WE pretreatment prevent inflammatory response induced by APAP.

### 3.2 AR.WE mitigated APAP-induced liver injury in the treatment experiments

**FIGURE 3:**
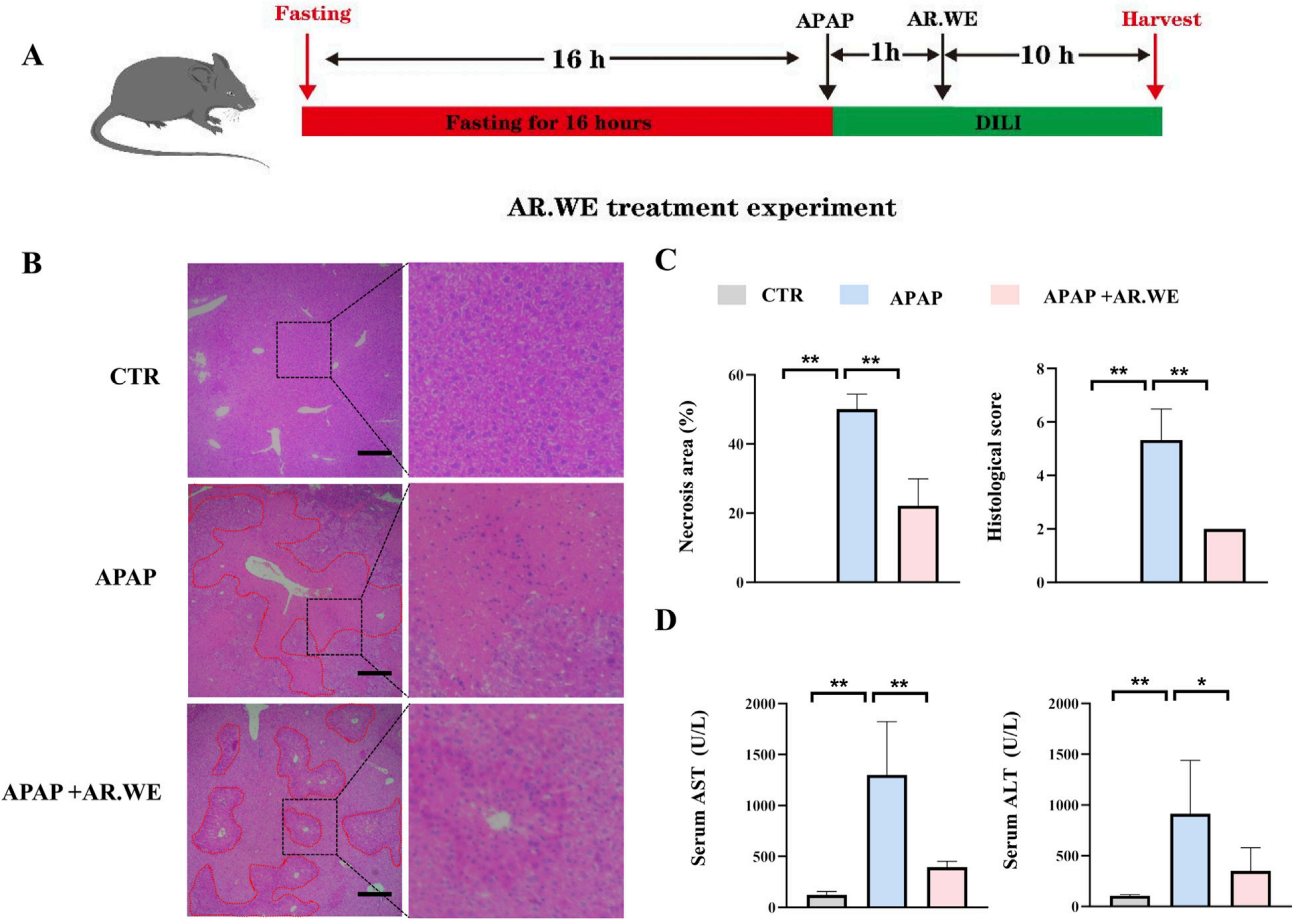
AR.WE can treat APAP-induced hepatotoxicity. **(A)** The time schedule fothe AR.WE for the treatment of APAP-induced liver injury. After fasting for 16 h, the mice were given APAP (300 mg/kg) to induce acute liver injury. After 1 h, a single dose of AR.WE (250 mg/kg) was administered intragastric. Then, blood and liver samples were collected after 10 h **(B)** H&E staining of mouse liver sections after treatment with AR.WE The dotted line indicates central lobular necrosis. Scale bars, 100 μm, n = 3. **(C)** Percentage of necrotic areas (left) and liver histological scores (right), n = 3. **(D)** Serum ALT (left) and AST (right) levels, n = 5–6. All group data subjected to statistical analysis were repeated in at least three independent experiments. The data are presented as mean ± SEM. *p < 0.05, **p < 0.01.

**FIGURE 4:**
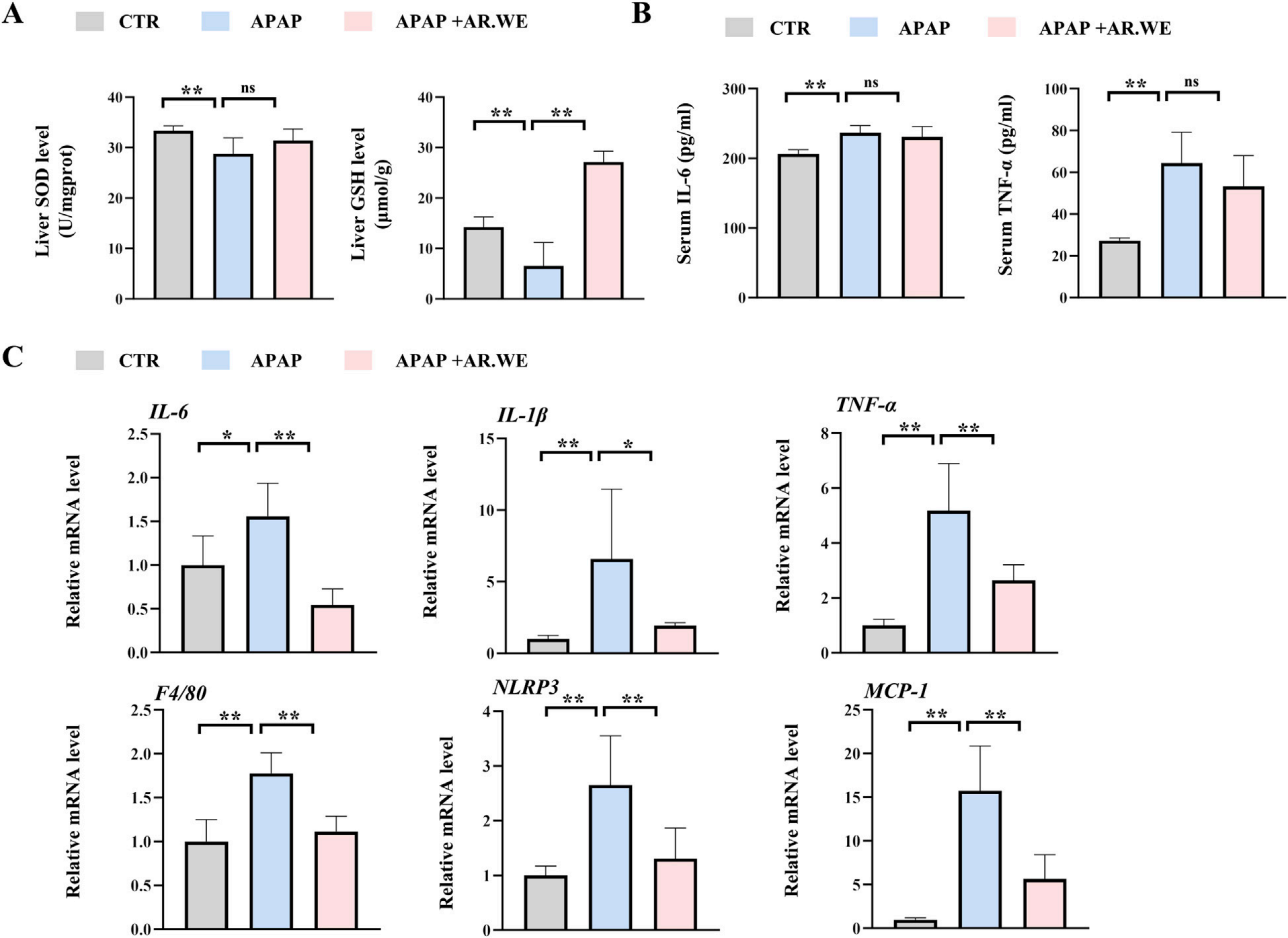
AR.WE can treat APAP-induced inflammation and oxidative stress. **(A)** Elisa analysis of serum IL-6 (left) and TNF-α (right), n = 5–6. **(B)** The level of liver SOD (left) and GSH (right), n = 5–6. **(C)** RT-PCR analysis of *IL-6, IL-1β, TNF-α, F4/80, NLRP3, MCP-1*, n = 5–6. All group data subjected to statistical analysis were repeated in at least three independent experiments. The data are presented as mean ± SEM. *p < 0.05, **p < 0.01.

Our previously results have shown that pretreatment of AR.WE for 3 days significantly prevent against APAP-induced liver injury, whether this herb is effective against the liver damage that has been caused is still unclear. Thus, mice were gavaged with AR.WE at 1 h after APAP administration, and were sacrificed 10 hour later (Figure 3A). Similarly, AR.WE treatment after APAP administration significantly reduced pathological changes and necrotic area of mouse liver, as revealed by H&E analysis (Figures 3B, C). Meanwhile, the liver function markers ALT and AST were also improved by AR.WE treatment (Figure 3D). Moreover, we found that AR.WE treatment significantly increased hepatic SOD levels and decreased the activity of hepatic GSH compared to the control group, therefore play an important role in antioxidant defense and stress resistance (Figure 4A). We then examined serum pro-inflammatory cytokine expression levels and the mRNA expression of genes related to inflammatory response. Consistently, APAP-induced inflammatory response in liver and circulation was significantly abolished by AR.WE (Figures 4B, C). Thus, we may conclude that AR.WE treatment after APAP administration mitigated APAP-induced liver injury.

### 3.3 ARPs preconditioning effectively prevented APAP-induced liver injury

**FIGURE 5:**
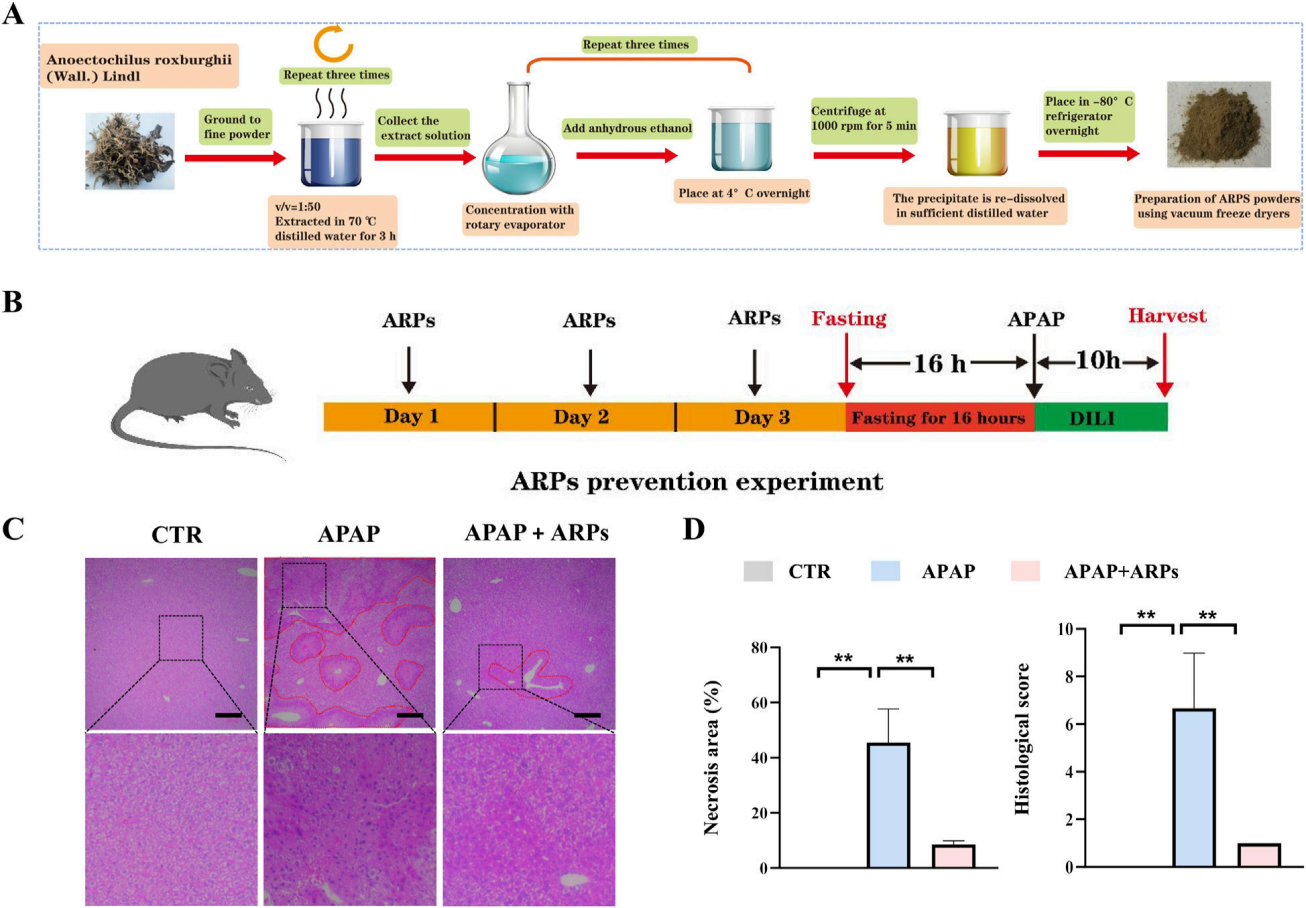
ARPs can prevent APAP-induced hepatotoxicity. **(A)** Flowchart for extracting ARPs. **(B)** The time schedule for ARPs for the prevention of APAP-induced liver injury. Mice were gavaged with ARPs (250 mg/kg) for 3 days continuously, and fasting was initiated for 16 h after the end of gavage on the third day. Acute liver injury was induced by administration of 300 mg/kg APAP at the end of fasting. Then, blood and liver samples were collected after 10 h **(C)** H&E staining of mouse liver sections after treatment with AR.WE. The dotted line indicates central lobular necrosis. Scale bars, 100 μm, n = 3. **(D)** Percentage of necrotic areas (left) and liver histological scores (right), n = 3. All group data subjected to statistical analysis were repeated in at least three independent experiments. The data are presented as mean ± SEM. *p < 0.05, **p < 0.01.

**FIGURE 6:**
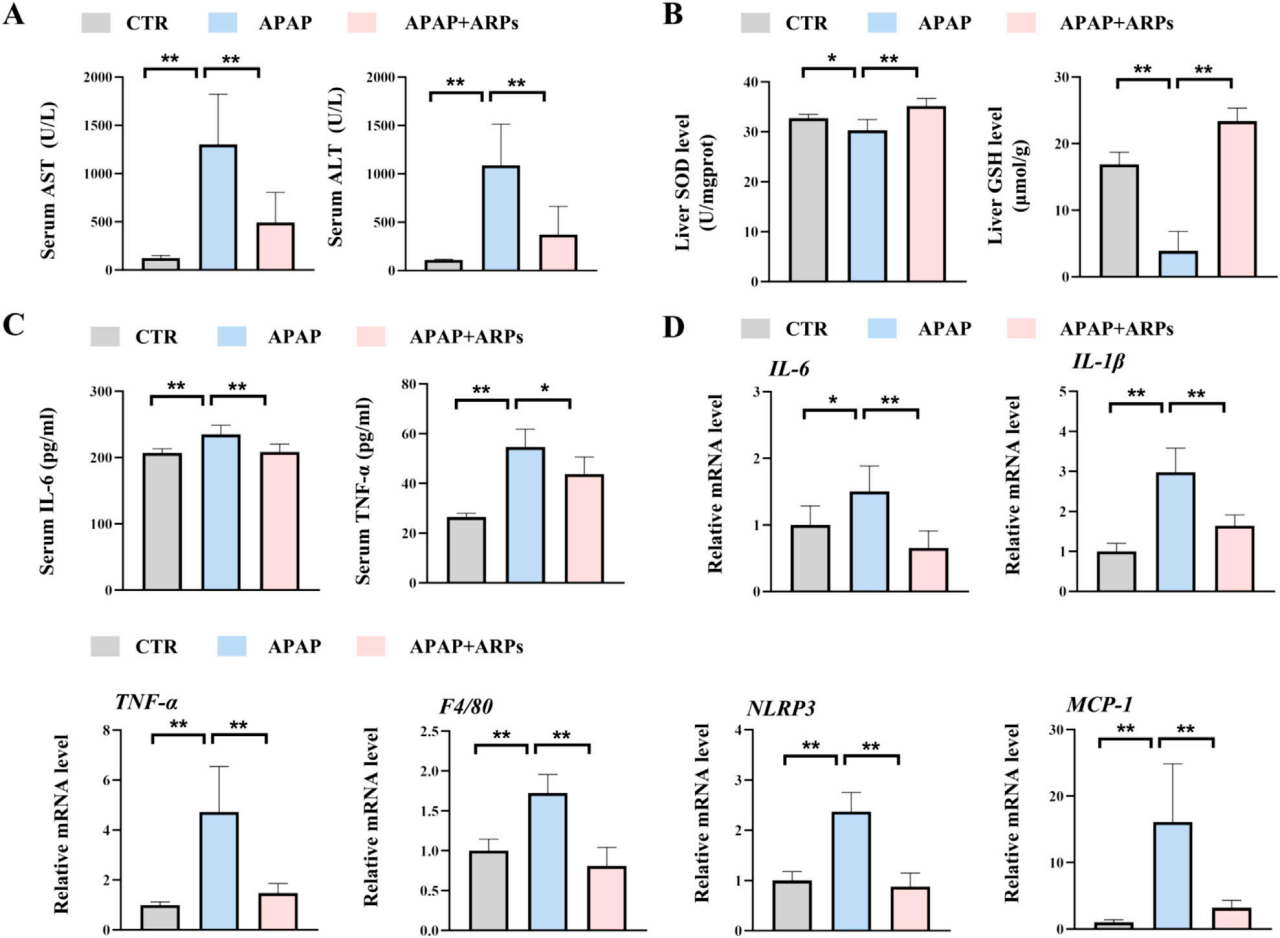
ARPs can prevent APAP-induced inflammation and oxidative stress. **(A)** Serum ALT (left) and AST (right) levels, n = 5–6. **(B)** ELISA analysis of serum IL-6 (left) and TNF-α (right), n = 5–6. **(C)** The levels of liver SOD (left) and GSH (right). **(D)** RT-PCR analysis of *IL-6, IL-1β, TNF-α, F4/80, NLRP3, MCP-1*, n = 5–6. All group data subjected to statistical analysis were repeated in at least three independent experiments. The data are presented as mean ± SEM. *p < 0.05, **p < 0.01, ns *p* ≥ 0.05.

ARPs is the main water-soluble component of AR, have been proved to play a significant role in antitumor, anti-inflammatory, antivirus, antihyperglycemic, anti-aging and anticoagulant activities (Liu et al., 2020). We then performed experiment to determine whether ARPS was the main component of AR.WE that exerts hepatoprotective effects. Polysaccharides from AR were usually extracted by the method of water extract and alcohol precipitate, followed by protein removal using the Sevag method, as shown in our previous research (Figure 5A). Before APAP administration, Mice were pretreated with ARPs at a dose of 250 mg/kg for 3 days (Figure 5B). As revealed by H&E analysis, ARPs preconditioning for 3 days ameliorated APAP-induced liver injury, much less hepatocellular injury and necrosis was observed in ARPs-treated group, indicating the protection of ARPs against APAP-induced liver toxicity (Figures 5C, D). Meanwhile, ARPs treatment reduced both ALT and AST levels significantly (Figure 6A). In addition, ARPs preconditioning significantly improved hepatic SOD and GSH activities compared to the APAP group (Figure 6B). Then, RT-PCR and ELISA assays were conducted to detect the expression change of the angiogenic factor and inflammatory factors at the mRNA levels and protein levels. ARPs-treated mice also exhibited significantly decreased pro-inflammatory factor expression levels which were consistent with the previous experimental results (Figures 6C–E). The above findings indicated that ARPs pretreatment effectively prevented acute liver damage induced by APAP.

### 3.4 ARPs alleviated APAP-induced inflammatory response in HepG2 cell lines

**FIGURE 7:**
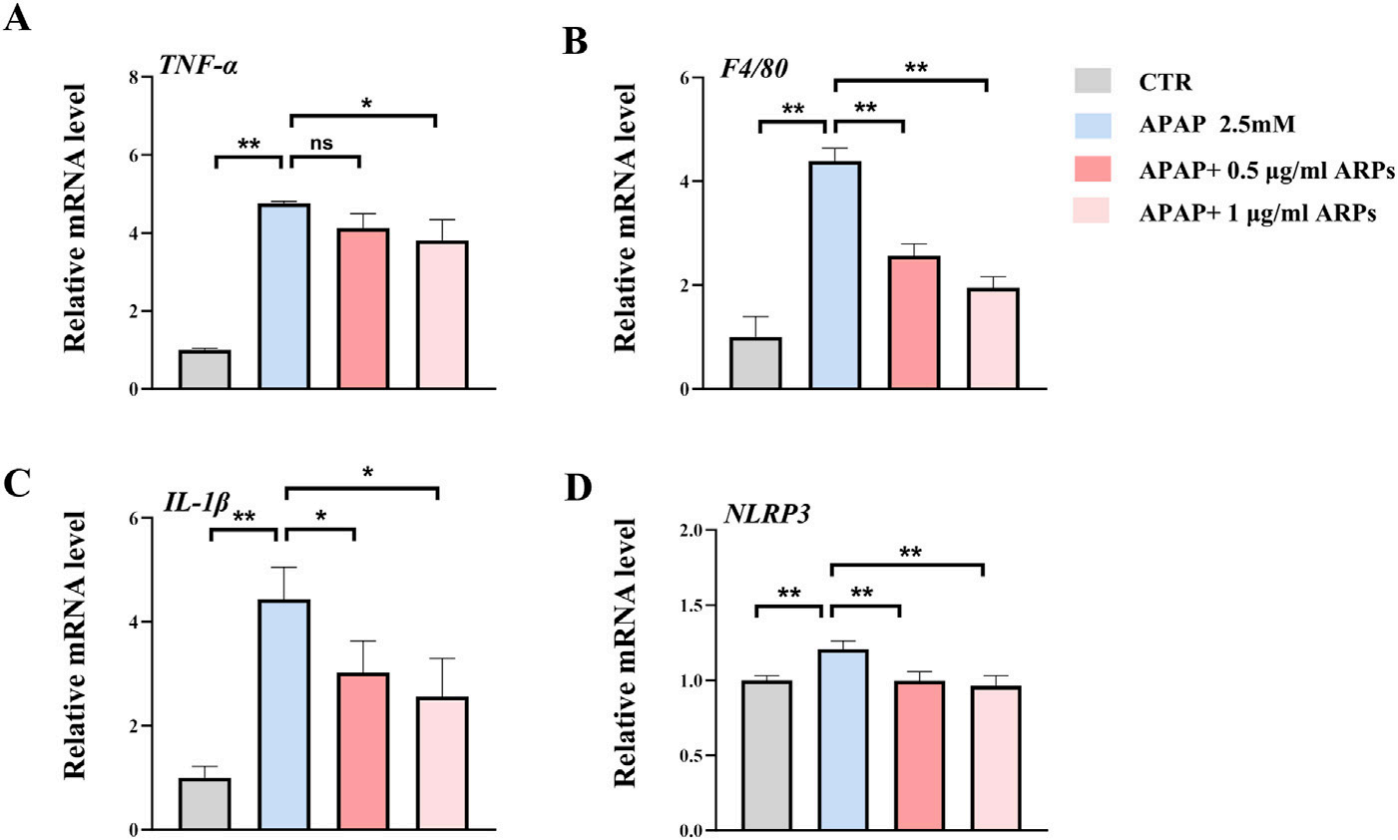
ARPs ameliorates APAP-induced inflammation in HEPG2 cells. APAP (2.5 mM) was given after pretreatment with ARPs (0.5 µg/ml and 1 µg/ml) for 1 h, then stimulated for 24 h before subsequent experiments. **(A–D)** RT-PCR analysis of *IL-1β, TNF-α, F4/80, NLRP3,* n=3. The data are presented as mean ± SEM. *p < 0.05, **p < 0.01, ns *p* ≥ 0.05.

To further test the potential effect of ARPs on hepatocytes, we performed *in vitro* experiment. HepG2 cell lines were preconditioned with ARPs for 1 h and then stimulated with APAP for 24 h. As revealed by RT-PCR analysis, ARPs preconditioning significantly upregulated genes involved in inflammation such as TNF-α, F4/80, and il-1β in a dose-dependent manner (Figure 7). The NLRP3 inflammasome is a cytosolic complex for early inflammatory responses, increasing evidence shows that the NLRP3 inflammasome is activated in APAP-induced liver injury in mice. Consistently, APAP exposure significantly increased the expression of NLRP3 in HepG2 cell, while the treatment of ARPs effectively reversed this phenotype (Figure 7). Thus, these *in vitro* results further verified the hepatoprotective effect in APAP-induced liver injury.

### 3.5 DIA-based quantitative proteomic analysis of mouse liver

**FIGURE 8:**
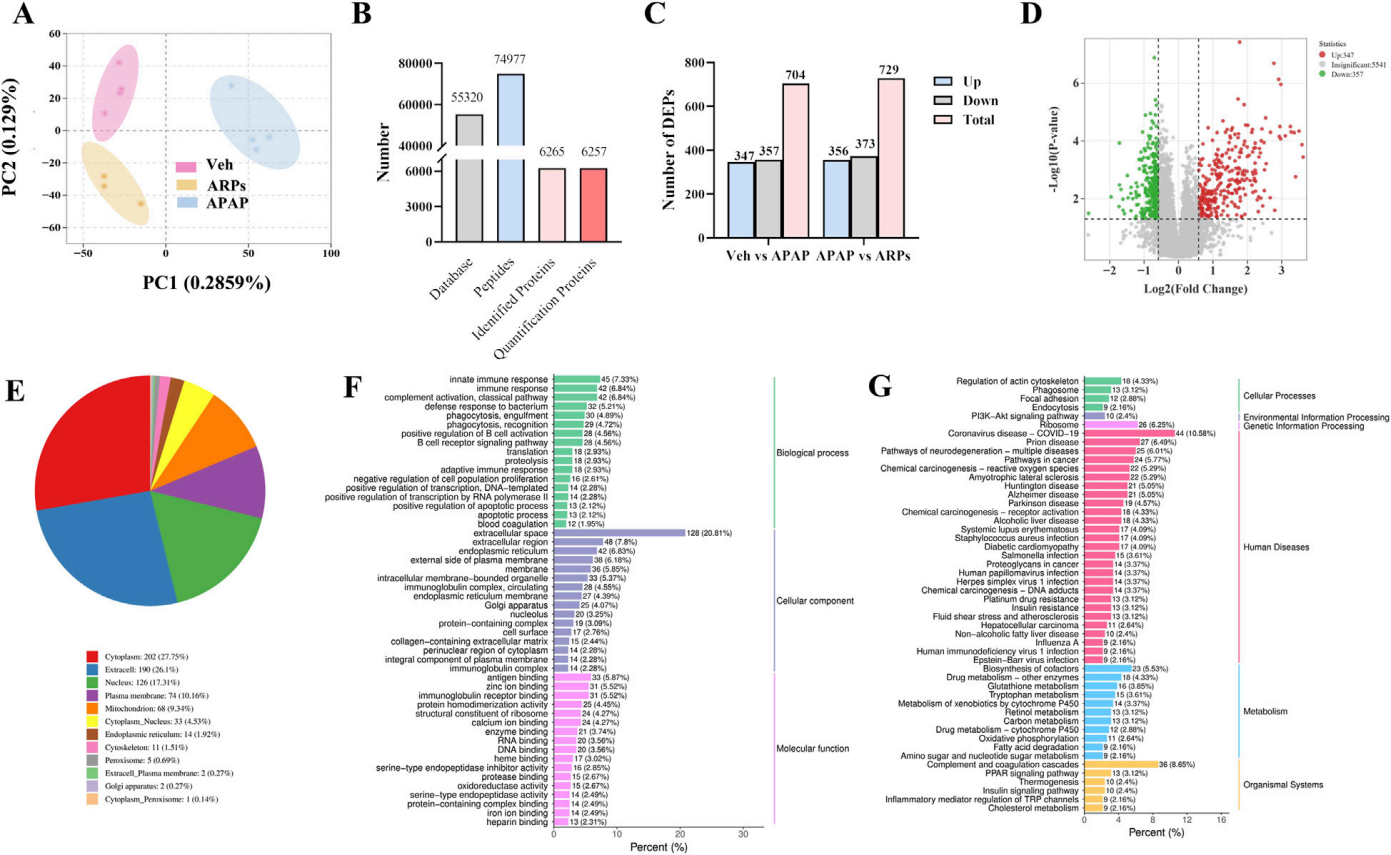
DIA-based quantitative proteomic analysis of mouse liver. **(A)** Principal Componen Analysis (PCA) results. The horizontal and vertical coordinates represent the first and second principal components, respectively, and the percentage in parentheses represents the contribution of the principal component to the sample variance; each point in the graph represents a sample, and different colours represent different groups of samples. **(B)** Statistical map of identification results. **(C)** The number of DEPs in Veh group vs APAP group, APAP group vs ARPs group, Veh group vs ARPs group. **(D)** Volcano plots of DEPs in APAP group vs ARPs group. Red points represented upregulated proteins and blue points represented downregulated proteins. **(E)** Pie graph of results of subcellular localization of differentially expressed proteins. **(F)** Bar graph of GO enrichment analysis of differentially expressed proteins. Horizontal coordinates represent the percentage of the number of differentially expressed proteins annotated to the entry; vertical coordinates represent the name of the GO entry. **(G)** Bar graph of the classification of the differentially expressed protein KEGG. The horizontal coordinate represents the ratio of differentially expressed proteins annotated to the pathway to the total number of proteins with annotations, and the vertical coordinate represents the name of the KEGG pathway.

To further explore the potential mechanisms by which ARPs ameliorates liver injury, we conducted a Data-independent acquisition (DIA)-based quantitative proteomics research. PCA analysis showed that the three patterns were distinct (Figure 8A). In total, 74,977 peptide fragments and 6,257 quantitative proteins were identified (Figure 8B). A total of 729 differentially expressed proteins (DEPs) (356 upregulated proteins and 373 downregulated proteins) were found between the APAP and ARPs groups (Figure 8C). The DEPs screening criteria was as follows: fold change (FC) ≥1.5 or ≤0.67 and P < 0.05. Volcano plot showing differential expressed genes between APAP and ARPs groups (Figure 8D).

**FIGURE 9:**
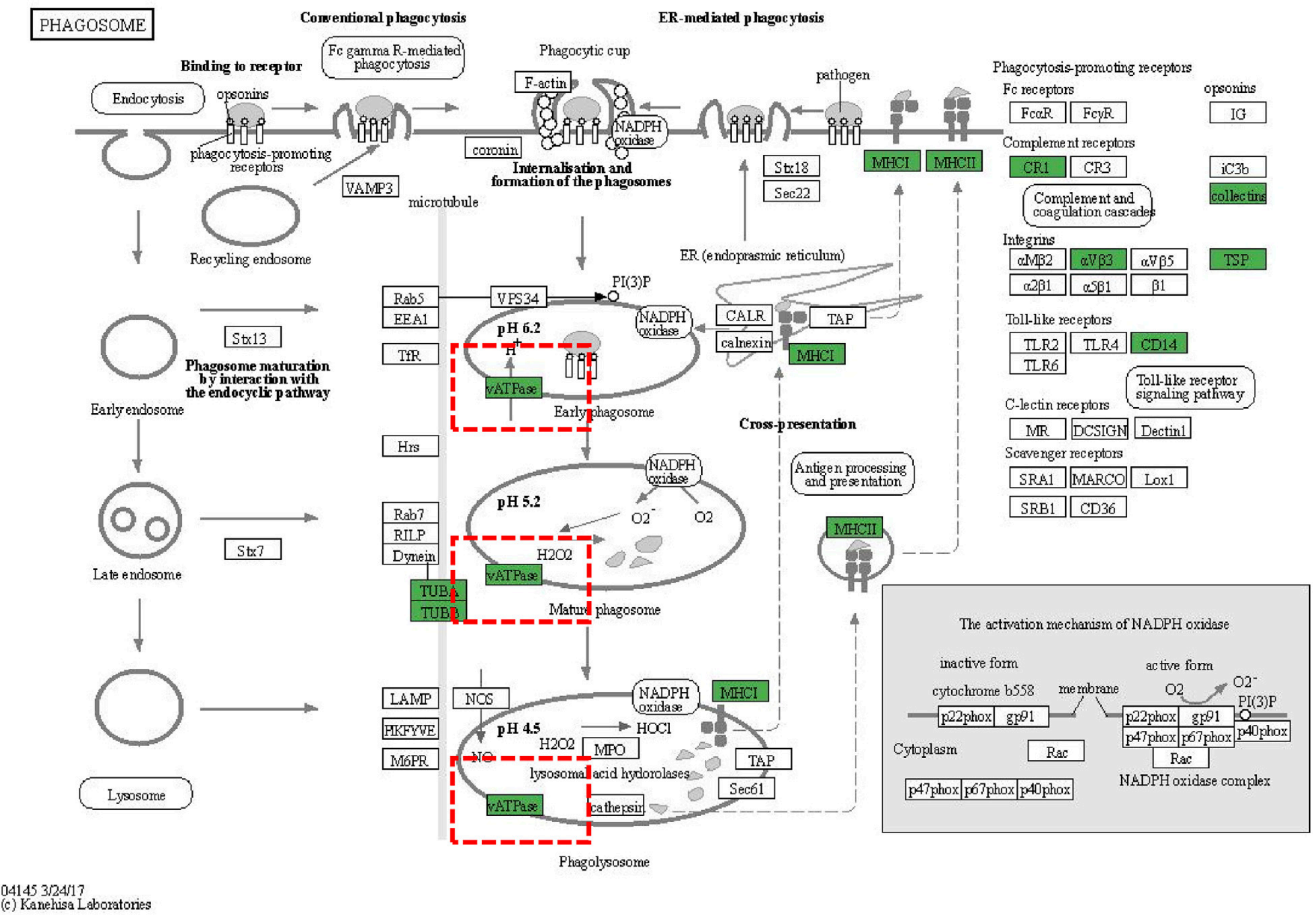
KEGG pathway diagrams of DEGs. KEGG pathway diagrams of DEGs. Green represents downregulation.

We conducted subcellular localization of DEPs between the APAP group and the ARPs group and demonstrated the top 12 most enriched. In the first place was the cytoplasm, indicating that 27.75% of DEPs were located in the cytoplasm, followed by extracell (26.1%), nucleus (17.31%), and plasma membrane (10.16%), respectively (Figure 8E). Then Gene Ontology (GO) annotation and Kyoto Encyclopedia of Genes and Genomes (KEGG) enrichment were performed based on these DEPs. The GO annotation divides these DEPs into three sections: biological processes, cellular component, and molecular function. For biological processes, DEPs in the APAP and ARPs groups were mainly enriched in innate immune response (Figure 8F). For cell contents, DEPs were mainly enriched in extracellular components. In terms of molecular function, DEPs were mainly enriched in antigen binding (Figure 8F). KEGG enrichment revealed that these DEPs were most enriched in regulation of actin cytoskelenon, and phagosome pathway (Figure 8G). We further analysed the phagosome pathway based on animal model and research direction. KEGG pathway map demonstrated that vacuolar H^+^-ATPases (vATPases) played an important role in the phagosome pathway (Figure 9). We therefore speculated that downregulation of vATPase activity was a potential target of ARPs for the treatment of liver injury.

**FIGURE 10:**
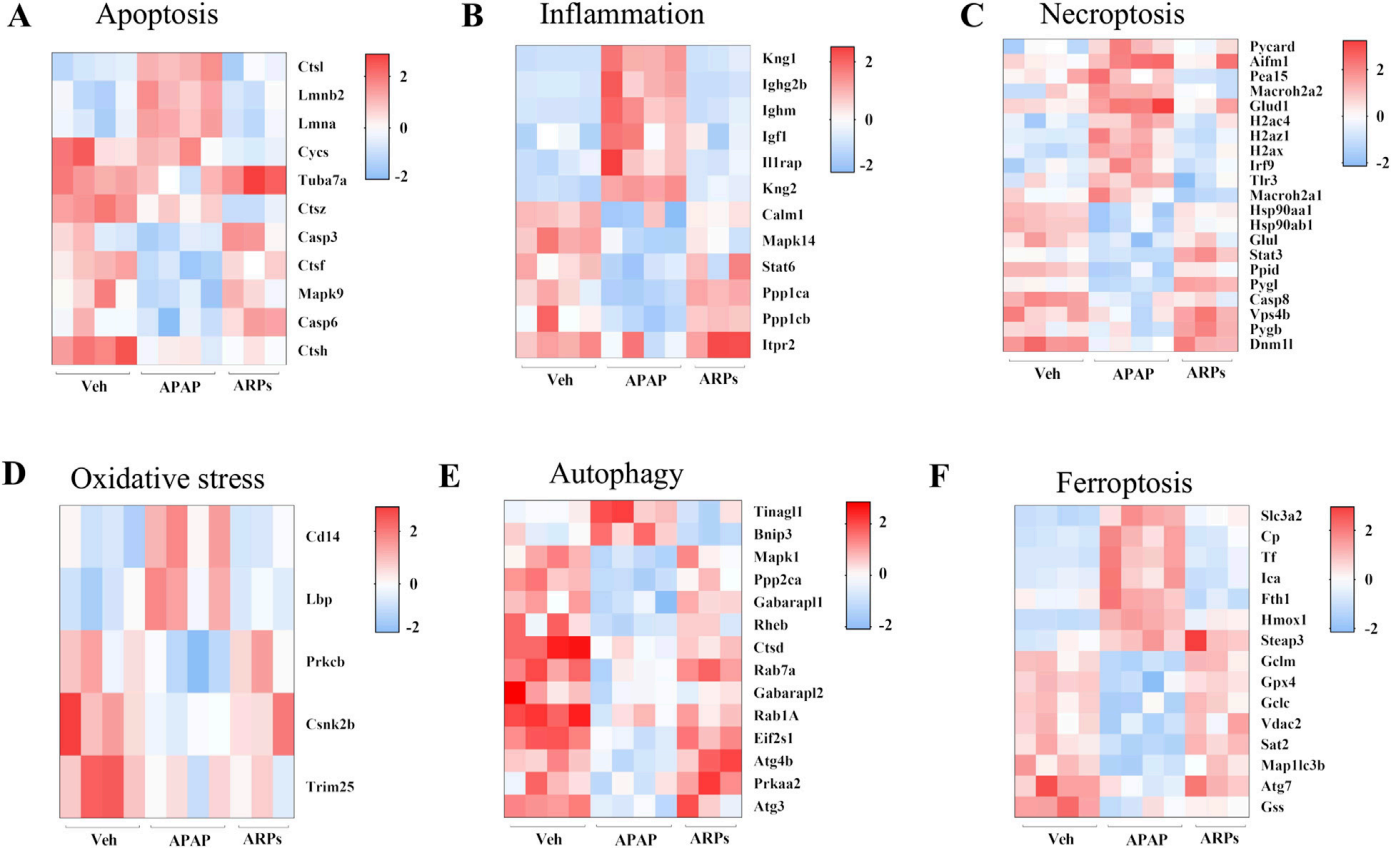
The protein levels involved in apoptosis, inflammation, necroptosis, and oxidative stress, autophagy, and ferroptosis **(A)** Heatmap of differential protein clustering involved in apoptosis. **(B)** Heatmap of differential protein clustering involved in inflammation. **(C)** Heatmap of differential protein clustering involved in necroptosis. **(D)** Heatmap of differential protein clustering involved in oxidative stress. **(E)** Heatmap of differential protein clustering involved in autophagy. **(F)** Heatmap of differential protein clustering involved in ferroptosis.

**FIGURE 11:**
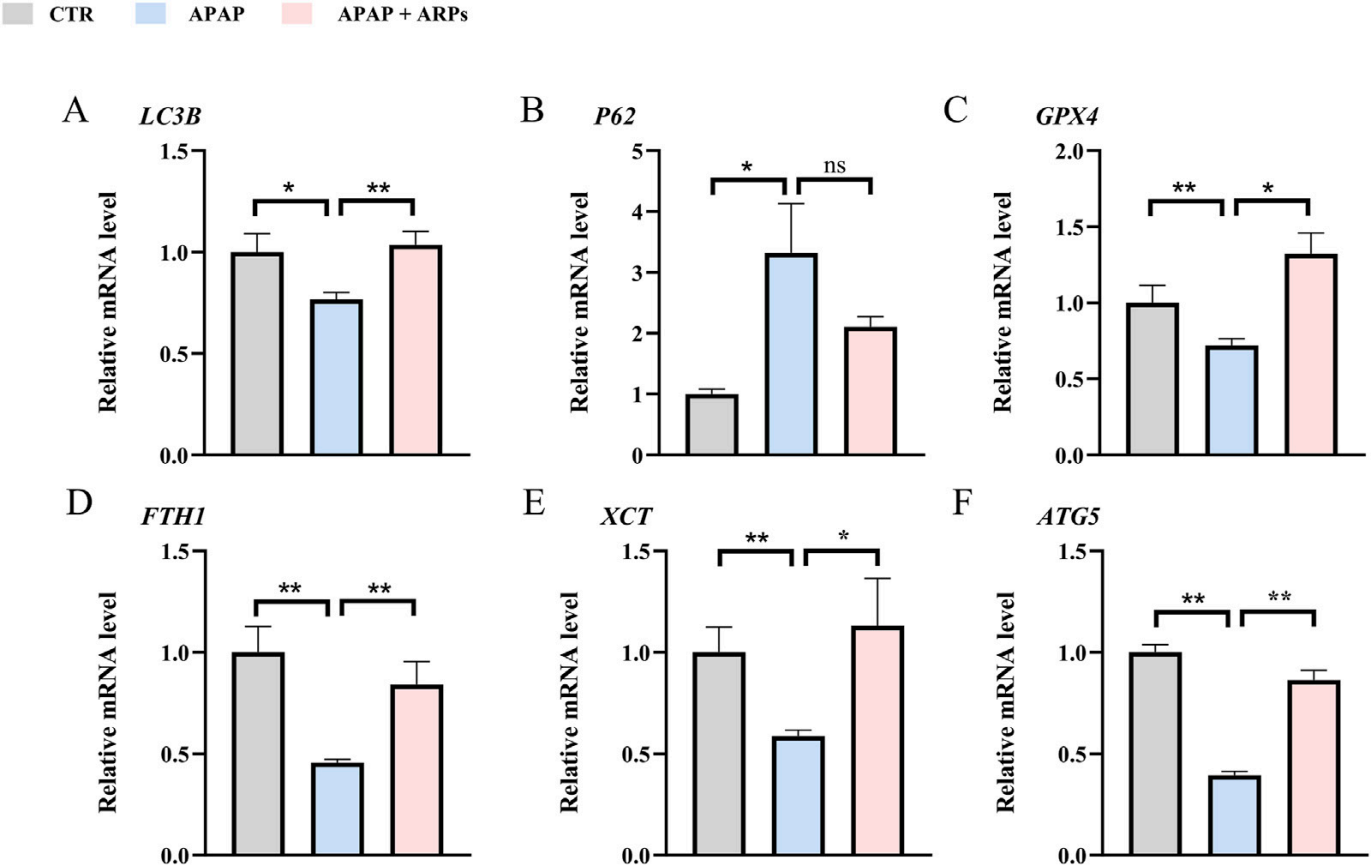
RT-PCR confirms that ARPs ameliorate liver injury by regulating genes involved in hepatocyte autophagy and ferroptosis. **(A)** RT-PCR analysis of *LC3B*; **(B)** RT-PCR analysis of P62; **(C)** RT-PCR analysis of GPX4; **(D)** RT-PCR analysis of FTH1; **(E)** RT-PCR analysis of XCT; **(F)** RT-PCR analysis of ATG5. The data are presented as mean ± SEM. *p < 0.05, **p < 0.01, ns *p* ≥ 0.05, n = 6.

We next assess the protein levels involved in apoptosis, inflammation, necroptosis, and oxidative stress, all of which important pathogenesis of APAP-induced liver injury. As shown in heatmaps, APAP exposure significantly upregulated the protein levels involved in pro-apoptosis, pro-inflammation, pro-necroptosis, and oxidative stress, while these phenotypes were prevented by ARPs treatment (Figures 10A–D). Hepatic autophagy is an essential mechanism for liver regeneration, and impaired autophagy is found to be related to dysfunction of various processes of hepatic regeneration (Wang et al., 2024). Thus, improving autophagy is an important therapeutic method to alleviate hepatic injury. Our data showed that ARPs could promote autophagy by transcriptionally activating autophagy-related genes, which indicated that ARPs may ameliorate APAP-induced liver injury by restoring autophagy (Figure 10E). APAP can aggravate oxidative stress and induce ferroptosis, a mode of cell death mediated by lipid peroxidation and cellular free iron when protective mechanisms such as glutathione peroxidase activity have been compromised (Jaeschke et al., 2021). In our data, ferroptosis-related genes were significantly regulated by ARPs treatment (Figure 10F). Consequently, we could speculate ARPs protects against drug-induced liver injury by inhibiting hepatocyte ferroptosis. To validate this conjecture, we performed RT-PCR analysis and found that Liver *P62* gene expression level was significantly higher in APAP group mice compared to control group (*P* = 0.01). After ARPs administration, *P62* gene expression level decreased (*P* = 0.17). Whereas, APAP stimulation resulted in decreased hepatic *LC3B* expression level in mice (*P* = 0.03). After ARPs administration, *LC3B* gene expression level increased (*P* = 0.004) (Figures 11A, B). In addition, the gene expression levels of *GPX4, FTH1, ATG5,* and *XCT* were reduced in the livers of mice in the APAP group compared to the control group, and the administration of ARPs reversed these trends (Figures 11C–F). All these data suggest that ARPs may ameliorate liver injury by regulating autophagy and ferroptosis.

## 4 Discussion

AR is a traditional medicinal plant in China with a variety of pharmacological effects such as liver protection, cancer prevention, treatment of cardiovascular disease and diabetes mellitus (Han et al., 2008; Ye et al., 2017). A study has reported the hypoglycaemic and antioxidant effects of water extract from AR on diabetic mice (Cui et al., 2013). It has also been shown that AR may alleviate aging and aging-related learning and memory impairment (Xiao et al., 2024). Meanwhile, Yan et al. found that AR ameliorates D-GalN/LPS-induced acute liver injury by modulating inflammatory vesicles. AR also showed a protective effect against isoniazid-induced drug-induced liver injury (Lin et al., 2024). In addition, multiple ameliorative effects of this herb on hepatic steatosis, oxidative stress, inflammatory infiltration, hepatocyte apoptosis and fibrosis in experimental non-alcoholic fatty liver disease (NAFLD) have been identified (Deng et al., 2022). Our study demonstrated that AR and its polysaccharide fractions can effectively ameliorate inflammatory infiltration and oxidative stress in acute liver injury induced by APAP, and the mechanism may be related to the hepatocyte autophagy, and downregulation of vATPase activity is likely to participate in this progress.

Oxidative stress has been implicated as an important factor in the development of various diseases such as liver disease, chronic kidney disease, neurodegenerative diseases, etc. (Samarghandian et al., 2017). Excess APAP leads to increased mitochondrial oxidative stress, generating large amounts of reactive oxygen species thereby damaging hepatocytes (Xu P. et al., 2022). It has been shown that AR protects against hydrogen peroxide-induced L02 cell damage, and the mechanism may involve oxidative stress. Moreover, Kinsenoside (an extract of AR) can ameliorate alcoholic liver injury in mice by reducing oxidative stress (Gao et al., 2021). Furthermore, Luo et al. found that AR protected the retinal pigment epithelium (RPE) from oxidative stress-induced apoptosis while reducing apoptosis-associated neovascularisation (Luo et al., 2018). In this study, we observed that AR exhibited significant anti-oxidative stress in APAP -induced liver injury, including increased GSH and SOD levels in the liver. These data were in line with the functional and phenotypic results described previously.

Chronic inflammation is a major cause of liver cirrhosis (Koyama and Brenner, 2017). Macrophage plays an important role in inflammation and liver injury (Saha et al., 2018). Activated Kupffer cells (hepatic Macrophages) secrete pro-inflammatory factors, including IL-6 and TNF-α, which ultimately lead to liver injury (Voican et al., 2011; Kurihara et al., 1997). APAP-induced hepatotoxicity involves sterile inflammation, macrophage modulates innate immune signaling stimulating factor (STING) mediated inflammation (Yang et al., 2023). Hsiao et al. found that AR alleviated acute inflammation by inhibiting pro-inflammatory cytokine (TNF-α, IL-6) release and enhancing SOCS - 3 mediated release of anti-inflammatory cytokines via the NF-κB signaling pathway (Hsiao et al., 2011). Uniformly, AR also ameliorated rheumatoid arthritis in rats by inhibiting the phosphorylation of IkB and P65 and down-regulating LPS-induced IL-1β and IL-6 (Guo et al., 2019). In this study, we found that AR reduced the expression of inflammatory factors such as TNF-α, IL-6, IL-1β, which highlight the therapeutic potential of AR for APAP -induced liver injury.

To further reveal the underlying mechanisms of AR amelioration of APAP-induced liver injury, we performed a DIA-based quantitative proteomics study. A total of 729 DEPs were identified between ARPs the APAP and group. In further biological analyses, KGEE enrichment showed that these DEPs were significantly enriched in the phagosome pathway. Phagosome is involved in the autophagy process and binds to lysosome in the final stage of autophagy, which becomes the final site of degradation (Zhou et al., 2023). Autophagy is closely linked to liver homeostasis (Gao et al., 2020). LC3 is a key molecule in the process of autophagy and can bind to the membrane surface of autophagosomes to become a marker of autophagosomes (Fan et al., 2021). P62 was the first selective autophagy receptor discovered and plays an important role in autophagosome formation (Huang et al., 2024). In this study, APAP exposure led to decreased LC3 expression and increased P62 expression, while ARPs inhibition these aberrant expressions. The modulation by AR on autophagy have been verified in other diseases models. Gao et al. found that AR activated AMPK-dependent autophagic pathway to attenuate liver injury in alcoholic liver-injured mice (Gao et al., 2021). In addition, Wang et al. demonstrated that AR upregulated GPX4 expression to ameliorate myocardial injury, a process that was accompanied by the regulation of Akt/Nrf2/HO-1 signalling (Wang et al., 2023).

Referring to the results of KEGG enrichment, we found that vATPase is involved in the biological process of phagosome. vATPase is a multisubunit protein complex that primarily functions to acidify lysosomes and promote autophagosomal degradation (Hooper et al., 2022). Meanwhile, vATPase activity is an indispensable component of the process of LC3 lipidation and distribution (Florey et al., 2015). In addition to its role in conventional autophagy, the researchers found that vATPase inhibits ferroptosis by activating TFEB-dependent SOD production, and may participate in iron death by affecting autophagy activity because the selective activation of autophagy mediates the degradation of antiferroptosis regulators, including ferritin (Chen F. et al., 2022). In conclusion, our study concluded that AR reduces oxidative stress and inflammatory infiltration by regulating vATPase activity and thus participating in the autophagic process and ferroptosis.

There are some limitations in our study. Firstly, although we found that AR can ameliorate APAP-induced liver injury, it has only been validated in mice due to the conditions. Therefore, clinical trials are needed to assess the effectiveness and safety of AR in humans. Second, AR contains a variety of bioactive components, including polysaccharides, glycosides, steroids, triterpenes, amino acids, and alkaloids. ARPs, one of the main components of AR, not only reduce blood glucose levels in diabetic mice by improving glucolipid metabolism, enhancing immunoprotection and reducing oxidative stress (Zhang et al., 2015). Although we demonstrated that ARPs can prevent liver injury, we cannot rule out that there are other components that play a role. Further experiments are needed in the future to determine the key active components of AR for the treatment of liver injury. Thirdly, the gut-liver axis plays an essential role in the pathogenesis of drug-induced liver injury (Chen Q. W. et al., 2022; Lu et al., 2024), and it has been demonstrated that many natural polysaccharides can improve the intestinal flora diversity (Liu et al., 2024; Li et al., 2023b; Xu et al., 2023; Fu et al., 2023). Given that ARPs are administered orally, and we can not rule out the effect of microbial flora and its metabolites such as short-chain fatty acids (SCFAs), which have been proven to act as molecular regulators in multiple liver diseases (Zhang et al., 2021).

## 5 Conclusion

In conclusion, we acknowledge that this study has some limitations, including the lack of human clinical trial data and the further investigation of detailed molecular mechanisms.

While this research also preliminarily clarified that AR as a promising new treatment for drug induced liver injury. This will broaden the potential applications of AR in liver diseases.

## Data Availability

The datasets presented in this study can be found in online repositories. The names of the repository/repositories and accession number(s) can be found in the article/Supplementary Material.

## Abbreviations

DILI, Drug-induced liver injury; AR, Anoectochilus roxburghii (Wall.); ARPs, Anoectochilus roxburghii polysaccharides; APAP, Acetaminophen; AR.WE, water extract of Anoectochilus roxburghii (Wall.); NAPQI, n-acetyl-p-benzoquinone-imine; GSH, glutathione; NAC, n-acetylcysteine; GPC, gel permeation chromatography; ELISA, Enzyme-Linked Immunosorbent Assay; SOD, Superoxide Dismutase; DEPs, differentially expressed proteins; NAFLD, non-alcoholicfatty liver disease; RPE, retinal pigment epithelium; STING, innate immune signaling stimulating factor; (v-ATPases), vacuolar H^+^-ATPases; DIA, Data-independent acquisition; HPLC, High-performance liquid chromatography; IAM, iodoacetamide; RPE, retinal pigment epithelium; GO, Gene Ontology; KEGG, Kyoto Encyclopedia of Genes and Genomes.
