# Supplementary material for: DIA-based quantitative proteomics explores the mechanism of amelioration of APAP-induced liver injury by anoectochilus roxburghii (Wall.) Lindl
Source: Front Pharmacol. 2025 Mar 26;16:1508290. doi: 10.3389/fphar.2025.1508290 (PMC11979217; doi:10.3389/fphar.2025.1508290)
Supplement: Supplementary file 1 [file Table1.docx]

**Supplementary Table 1. List of primers sequences for qRT-PCR analysis.**

| Gene | Fwd (5’-3’) | Rev (5’-3’) |
| --- | --- | --- |
| mTNF-α | GGCGGTGCCTATGTCTCA | AGGGTCTGGGCCATAGAA |
| mIL-1β | GCAACTGTTCCTGAACTCAACT | ATCTTTTGGGGTCCGTCAACT |
| mIL-6 | TAGTCCTTCCTACCCCAATTTCC | TTGGTCCTTAGCCACTCCTTC |
| mF4/80 | TGACTCACCTTGTGGTCCTAA | CTTCCCAGAATCCAGTCTTTCC |
| mNLRP3 | ATTACCCGCCCGAGAAAGG | TCGCAGCAAAGATCCACACAG |
| mMCP-1 | TGGCTCAGCCAGATGCAGT | CCAGCCTACTCATTGGGATCA |
| hIL-1β | ATGATGGCTTATTACAGTGGCAA | GTCGGAGATTCGTAGCTGGA |
| hTNF-α | CCTCTCTCTAATCAGCCCTCTG | GAGGACCTGGGAGTAGATGAG |
| hF4/80 | CAGCGTTCTGGACAAAGTGTG | CGGAGTGATATTTGCTGAGGGT |
| hNLRP3 | GATCTTCGCTGCGATCAACAG | CGTGCATTATCTGAACCCCAC |
